# Therapeutic strategies for KRAS G12C-mutant non-small cell lung cancer: from bench to bedside and beyond

**DOI:** 10.3389/fphar.2025.1704347

**Published:** 2026-01-26

**Authors:** Renjie Huang, Xian Gong, Jianting Du, Guobing Xu, Jiekun Qian, Guoliang Liao, Yuxing Lin, Maojie Pan, Bin Zheng, Wenjie Yuan, Qinzhao Huang, Chun Chen, Zhang Yang

**Affiliations:** 1 Department of Thoracic Surgery, Fujian Medical University Union Hospital, Fuzhou, Fujian, China; 2 Department of Thoracic Surgery, Fuzhou Pulmonary Hospital of Fujian, Fuzhou, Fujian, China; 3 Key Laboratory of Cardio-Thoracic Surgery, Fujian Medical University, Fuzhou, Fujian, China

**Keywords:** combination strategies, drug resistance, KRAS G12C, non-small cell lung cancer (NSCLC), targeted therapy

## Abstract

KRAS is one of the most frequently mutated oncogenes in non-small cell lung cancer (NSCLC), particularly in lung adenocarcinoma, with mutation rates ranging from 15% to 25%. Historically considered “undruggable,” KRAS has recently become a viable therapeutic target with the development of selective KRAS G12C inhibitors such as sotorasib (AMG510) and adagrasib (MRTX849). These inhibitors have demonstrated promising clinical efficacy; however, their effectiveness is frequently limited by the emergence of resistance mechanisms. This review provides a comprehensive analysis of KRAS G12C structural biology, its role in oncogenic signaling, and the challenges associated with targeted therapy. We discuss the mechanisms of intrinsic and acquired resistance, current monotherapy limitations, and the rationale for combination strategies aimed at overcoming resistance. Additionally, we explore future therapeutic perspectives, including novel inhibitors, combination regimens, and emerging precision medicine approaches, to optimize treatment outcomes for patients with KRAS G12C-mutant NSCLC.

## Highlights

• First comprehensive synthesis of monotherapy and combination strategies for KRAS G12C-mutant NSCLC, integrating latest clinical data on sotorasib, adagrasib, and next-generation inhibitors.
• Dissects three pillars of resistance—secondary KRAS mutations, RTK–MEK/PI3K bypass signaling, and histological transformation—offering mechanistic frameworks to guide rational combinations.
• Proposes a “vertical + horizontal + immuno-oncology” triad: vertical blockade of the RAS–MAPK axis (KRAS + SHP2/MEK/mTOR), horizontal co-targeting of PI3K–AKT, JAK–STAT, and YAP/TAZ pathways, plus synergistic PD-(L)1 blockade.
• Outlines precision-medicine roadmap leveraging co-mutation profiling (STK11/KEAP1), liquid-biopsy monitoring, and AI-driven trial design to optimize dosing and prolong durable responses.

## 1 Introduction

Lung cancer remains the leading cause of cancer-related mortality worldwide, with non-small cell lung cancer (NSCLC) accounting for approximately 85% of cases (Bray et al., 2024; Molina et al., 2008). Identifying and characterizing genetic mutations in NSCLC is crucial for guiding targeted therapies. A prime example is the third-generation epidermal growth factor receptor (EGFR) inhibitor osimertinib, which has significantly improved overall survival (OS) and progression-free survival (PFS) in metastatic and adjuvant treatment settings (Soria et al., 2018; Tsuboi et al., 2023). Among oncogenic drivers in NSCLC, KRAS mutations occur in approximately 25%–30% of cases, predominantly in lung adenocarcinomas. Historically, KRAS was considered “undruggable” due to its smooth protein surface and high affinity for guanosine triphosphate (GTP) and guanosine diphosphate (GDP), which hindered direct pharmacological targeting. However, groundbreaking discoveries have led to the development of small-molecule inhibitors specifically targeting the KRAS G12C mutation, such as sotorasib and adagrasib (Canon et al., 2019; Hallin et al., 2020). These inhibitors covalently bind to the mutant cysteine at codon 12, locking KRAS in its inactive GDP-bound state and thereby suppressing downstream oncogenic signaling.

Despite the clinical success of KRAS G12C inhibitors, their long-term efficacy is often limited by tumor heterogeneity and acquired resistance mechanisms. Resistance can arise through secondary KRAS mutations, bypass signaling activation, and histological transformations that enable tumor cells to evade targeted inhibition (Ryan et al., 2020; Awad et al., 2021; Tanaka et al., 2021; Li et al., 2022b). Consequently, monotherapy with KRAS G12C inhibitors may not provide durable disease control (Lindsay et al., 2021; Hata and Shaw, 2020).

To overcome these challenges, combination therapeutic strategies are under active investigation. These approaches integrate KRAS G12C inhibitors with immunotherapy, chemotherapy, or other targeted agents to enhance efficacy, delay resistance, and improve patient outcomes. The evolution of these treatment strategies underscores the shift toward personalized and precision oncology for KRAS G12C-mutant NSCLC.

## 2 KRAS G12C mutation and non-small cell lung cancer: prevalence, biology, and oncogenic signaling

KRAS mutations are among the most common oncogenic alterations in non-small cell lung cancer (NSCLC), occurring in approximately 25%–30% of cases (Garcia-Robledo et al., 2022). Among these, the KRAS G12C mutation—characterized by a glycine-to-cysteine substitution at codon 12—accounts for nearly 40% of all KRAS mutations, followed by G12V (21%) and G12D (17%) (Dogan et al., 2012). This mutation is most frequently observed in lung adenocarcinoma (32%) and is relatively uncommon in squamous NSCLC (∼5%) (Martin et al., 2013). The prevalence of KRAS mutations varies by population, with a higher frequency in Western countries, particularly among White individuals, and a lower incidence in Asian populations. Additionally, a strong correlation exists between KRAS G12C mutations and smoking history, as this alteration is significantly more prevalent in long-term smokers, whereas KRAS G12D mutations are more commonly found in never-smokers (Nassar et al., 2021; Adderley et al., 2019).

KRAS plays a pivotal role in regulating intracellular signaling pathways that control cell growth, differentiation, and survival (Moore et al., 2020; Drosten and Barbacid, 2020). As a member of the Ras GTPase family, the KRAS protein consists of a G domain and a hypervariable region (HVR) (Cox et al., 2014). The G domain includes three key structural elements—Switch I, Switch II, and the P-loop—that govern KRAS activity (Vetter and Wittinghofer, 2001). Oncogenic KRAS mutations disrupt its intrinsic GTPase activity, leading to continuous activation of downstream signaling cascades that drive tumorigenesis (Friedlaender et al., 2020; Ferrer et al., 2018).

**FIGURE 1:**
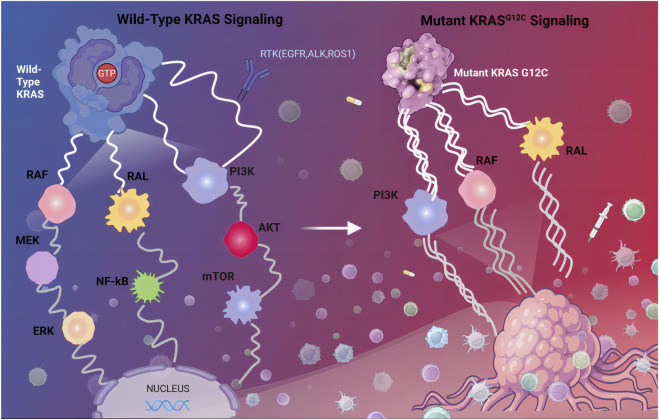
Wild-Type KRAS Signaling and Mutant KRAS G12C Signaling. KRAS binds to guanine nucleoside triphosphate (GTP), the KRAS protein is activated and continuously transmitting activation signals to downstream pathway, including the MAPK signaling pathway, the PI3K signaling pathway, and the Ral-GEFs signaling pathway. Abbreviations: RTK, receptor tyrosine kinase; EGFR, epidermal growth factor receptor; PI3K, phosphoinositide 3-kinase.

The KRAS G12C mutation alters the conformational dynamics of the KRAS protein, impairing its ability to switch between the inactive (GDP-bound) and active (GTP-bound) states (Wiesweg et al., 2019). Under normal conditions, KRAS cycles between these two states, with GDP binding maintaining an inactive conformation and GTP binding triggering activation (Ryan and Corcoran, 2018). The G12C mutation, however, shifts the equilibrium toward a constitutively active GTP-bound state, resulting in persistent stimulation of oncogenic pathways such as RAF-MEK-ERK, PI3K-AKT-mTOR, and RalGEF (Moodie et al., 1993; Sjölander et al., 1991; Liu et al., 2019) (Figure 1). This aberrant signaling drives uncontrolled cell proliferation, enhances survival mechanisms, and promotes tumor progression through angiogenesis and metabolic reprogramming.

Advancements in understanding KRAS G12C biology have facilitated the development of selective inhibitors that covalently bind to the mutant cysteine within the Switch II pocket, locking KRAS in its inactive GDP-bound state. While these inhibitors have demonstrated promising clinical efficacy, intrinsic and acquired resistance mechanisms pose significant challenges, necessitating further research into combination therapies and novel therapeutic strategies.

## 3 Therapeutic strategies for KRAS mutant lung cancer

The Ras signaling pathway plays a critical role in tumorigenesis by accelerating the cell cycle, inhibiting apoptosis, promoting invasion and metastasis, and enhancing tumor cell viability. Given its oncogenic significance, extensive research has been devoted to targeting Ras-driven tumors, focusing on two primary strategies: direct KRAS inhibition and indirect modulation of upstream and downstream signaling pathways. Direct inhibition involves small-molecule covalent inhibitors like sotorasib (AMG 510) and adagrasib (MRTX849) (Nokin et al., 2024), which selectively target KRAS G12C by locking it in its inactive GDP-bound state, preventing downstream oncogenic signaling. Alternatively, indirect approaches aim to suppress Ras activity by inhibiting upstream regulators such as receptor tyrosine kinases (RTKs) or downstream effectors like MEK, PI3K, and mTOR (McCubrey et al., 2011).

**FIGURE 2:**
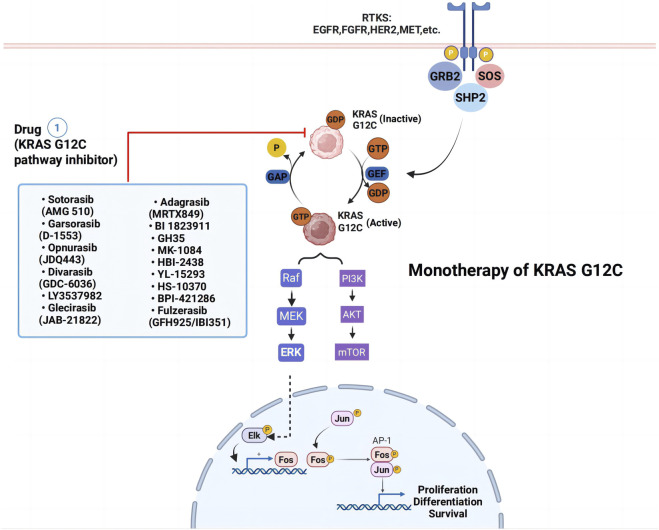
Monotherapy strategies for KRAS G12C-mutated non-small cell lung cancer. Blue box represent KRASG12C inhibitors. Abbreviations: RTK, receptor tyrosine kinase; EGFR, epidermal growth factor receptor; FGFR, Fibroblast Growth Factor Receptors; PI3K, phosphoinositide 3-kinase.

Historically, KRAS was considered “undruggable” due to its smooth, featureless structure and high affinity for GTP/GDP, which hindered competitive inhibition (Maurer et al., 2012; Pantsar, 2019). Consequently, treatment for KRAS-mutant lung cancer relied primarily on conventional chemotherapy and radiation, which demonstrated limited efficacy. More recently, immune checkpoint inhibitors (ICIs) have emerged as an effective alternative, particularly in combination regimens (Hames et al., 2016; Yagishita et al., 2015; Hallqvist et al., 2012; Negrao et al., 2021). Extensive research is underway to explore new therapeutic approaches for KRAS G12C mutations, including targeted small molecule inhibitors (Figure 2) and drug combinations. Notably, the identification of the Switch II pocket on KRAS G12C revolutionized drug development, enabling the design of covalent inhibitors that selectively target this mutant protein. This breakthrough has provided a promising therapeutic avenue for KRAS G12C-driven NSCLC, surpassing the limitations of traditional treatments (Ostrem and Shokat, 2016; Ostrem et al., 2013; Janes et al., 2018).

### 3.1 Monotherapy of KRAS G12C inhibitors

#### 3.1.1 Sotorasib (AMG-510)

Sotorasib (AMG 510) is an oral, selective, and irreversible KRAS G12C inhibitor that covalently binds to the mutant cysteine at codon 12, locking KRAS in its inactive GDP-bound state and preventing downstream oncogenic signaling (Fell et al., 2020; Pantsar, 2020; Saiki et al., 2019). Its isopropylmethylpyridine substituent efficiently occupies the Switch II pocket, particularly the H95 groove, inhibiting GDP dissociation and disrupting oncogenic signaling (Fell et al., 2020; Pantsar, 2020; Saiki et al., 2019). Preclinical studies have shown that sotorasib nearly completely suppresses ERK phosphorylation, a key component of the MAPK pathway, in KRAS G12C-mutant cell lines (Canon et al., 2019). The clinical efficacy of sotorasib was demonstrated in the Phase I/II CodeBreaK 100 trial (NCT03600883), in which 126 patients with previously treated KRAS G12C-mutant NSCLC received a 960 mg dose, achieving an objective response rate (ORR) of 37.1%, including a complete response (CR) rate of 3.2%, a disease control rate (DCR) of 80.6%, a median progression-free survival (PFS) of 6.8 months, and a median overall survival (OS) of 12.5 months (Skoulidis et al., 2021; Nakajima et al., 2022; Li et al., 2021). The efficacy of sotorasib in patients with central nervous system (CNS) metastases remains unclear, as the study excluded those with active brain metastases. Adverse events were mostly mild to moderate, with grade 3 treatment-related adverse events (TRAEs) occurring in 19.8% of cases, and no fatal TRAEs reported (De Langen et al., 2023; Issahaku et al., 2023; Negrao et al., 2023). The Phase III CodeBreaK 200 trial (NCT04303780) compared sotorasib to docetaxel in previously treated NSCLC patients with KRAS G12C mutations. While the trial met its primary endpoint, demonstrating a modest improvement in PFS (5.6 vs. 4.5 months) and a superior safety profile compared to docetaxel, no significant OS advantage was observed (10.6 vs. 11.3 months) (De Langen et al., 2023). Despite these results, concerns arose regarding the study design, early dropout rates, and potential bias in imaging assessments, leading the FDA to question whether CodeBreaK 200 provided sufficient evidence to support sotorasib’s clinical benefit. To further evaluate its efficacy, the CodeBreaK 201 trial (NCT04933695) is assessing sotorasib as a first-line treatment for stage IV KRAS-mutant NSCLC patients with PD-L1 tumor proportion scores (TPS) < 1% and/or STK11 co-mutations, focusing on ORR, DCR, PFS, and OS as primary clinical endpoints (De Langen et al., 2023). This biomarker-driven approach is further supported by subgroup analyses from CodeBreaK 100 and 200, which demonstrated that sotorasib efficacy was maintained regardless of STK11 status in KEAP1-wildtype tumors, while KEAP1 co-mutations were associated with poorer outcomes across all treatment arms (De Langen et al., 2023). By specifically targeting patients with PD-L1-low/STK11-mutant tumors who are less likely to benefit from immunotherapy, CodeBreaK 201 aims to establish a rational first-line role for sotorasib in a precisely defined population with high unmet medical need.

#### 3.1.2 Adagrasib (MRTX849)

Adagrasib (MRTX849) is another oral, selective KRAS G12C inhibitor that employs a similar mechanism of action as sotorasib, binding covalently to the mutant cysteine within the Switch II pocke (Hallin et al., 2020; Fell et al., 2020). Unlike sotorasib, adagrasib has a prolonged half-life, high oral bioavailability, extensive tissue distribution, and significant CNS penetration, making it particularly effective in treating brain metastases (Issahaku et al., 2023). In preclinical studies, adagrasib selectively targeted KRAS G12C, suppressing KRAS-dependent signaling and tumor growth (Hallin et al., 2020). The Phase I/II KRYSTAL-1 trial (NCT03785249) demonstrated an ORR of 45%, a DCR of 96%, a median PFS of 5.4 months, and a median OS of 11.4 months in pretreated NSCLC patients (Negrao et al., 2023b). Notably, adagrasib exhibited promising intracranial activity, with a confirmed intracranial ORR of 33.3% and a median intracranial response duration of 11.2 months in patients with CNS metastases (Jänne et al., 2022). Based on these results, the U.S. FDA granted accelerated approval for adagrasib on December 12, 2022, for the treatment of adult patients with locally advanced or metastatic NSCLC carrying KRAS G12C mutations who had received prior systemic therapy. Given that 27%–42% of NSCLC patients with KRAS G12C mutations present with CNS metastases at diagnosis, a factor associated with poor prognosis, adagrasib’s ability to penetrate the blood-brain barrier is particularly significant (Cui et al., 2020; Isaksson et al., 2023). A preliminary evaluation at the 2022 American Society of Clinical Oncology meeting showed significant central nervous system (CNS) penetration in untreated brain metastases, with a median intracranial PFS of 4.2 months (Sabari et al., 2022). The KRYSTAL-12 Phase III trial (NCT04685135), comparing adagrasib to standard chemotherapy in previously treated NSCLC patients, has validated its clinical efficacy. The primary analysis published in Lancet demonstrated that adagrasib significantly improved PFS and ORR over docetaxel, with a median follow-up of 9.4 months. The primary endpoint of PFS was 5.49 months for adagrasib vs. 3.84 months for docetaxel (HR = 0.58, 95% CI: 0.45-0.76, p < 0.0001), while ORR by blinded independent central review (BICR) was 31.9% vs. 9.2%, with a median duration of response (DOR) of 8.31 vs. 5.36 months, respectively (Lee and Nagasaka, 2024; Mok et al., 2024; Lee et al., 2024; Barlesi et al., 2025). However, adagrasib’s PFS of 5.5 months in KRYSTAL-12 closely mirrors the 5.6-month PFS of sotorasib in CodeBreaK 200, failing to surpass the anticipated 6-month benchmark, raising concerns about the durability of monotherapy. The Clinical trials of KRAS G12C small molecule inhibitor monotherapies in NSCLC are summarized in Table 1.

**TABLE 1:** Clinical trials of KRAS G12C small molecule inhibitor monotherapies in NSCLC.

Drug	Trial name	Clinical trials identifier	Sponsor	Phase	Efficacy data	References
Sotorasib (AMG 510)	CodeBreaK 200	NCT04303780	Amgen	III	ORR:28.1% DCR:82.5% mPFS:5.6 months	de-Langen et al. (2023)
Adagrasib (MRTX 849)	KRYSTAL-12	NCT04685135	Mirati Therapeutics	III	ORR:32% DOR:8.3 months	Mok et al. (2024)
JDQ443	KontRASt-01	NCT04699188	Novartis	Ib/II	ORR:41.7%	Cassier et al. (2023)
GDC-6036 (Divarasib)	​	NCT04449874	Genentech	I	mPFS:13.1	Sacher et al. (2023)
D-1553 (garsorasib)	​	NCT05383898	InventisBio	I/II	Phase I:ORR:40.5%, DCR:91.9%,mPFS:8.2 months; Phase II:ORR:50%,DCR:89%,mPFS:7.6 months	Li et al. (2023)
JAB-21822 (Glecirasib)	​	NCT05009329	Jacobio	II	ORR:47.9% DCR:86.3% mOS:13.6 months	Jian et al. (2022), Shi et al. (2024)
GFH925 (IBI351)	​	NCT05005234	GenFleet Therapeutics	II	ORR:49.1% DCR:90.5% mPFS:9.7 months	Zhou et al. (2024)
LY3537982 (olomorasib)	LOXO-RAS-20001	NCT04956640	Eli Lilly	I/II	ORR:39% DCR:73% mPFS:9.7 months	Heist et al. (2024)

NCT: national clinical trial; ORR: Objective Response Rate; DCR: Disease Control Rate; mPFS: Median progression-free survival; mOS: Median overall survival.

#### 3.1.3 Other KRAS G12C inhibitors

Several next-generation KRAS G12C inhibitors are in clinical development, aiming to enhance efficacy and overcome resistance. JAB-21822 (Glecirasib), LY3537982 (Olomorasib), JDQ443, GFH925 (IBI351), GDC-6036 (Divarasib), and D-1553 (Garsorasib) have demonstrated promising results in early-phase trials (Jian et al., 2022; Shi et al., 2024; Heist et al., 2024; Cassier et al., 2023; Zhou et al., 2024; Tran et al., 2023; Sacher et al., 2023; Li et al., 2023; Li et al., 2024a). The Phase I/II study of glecirasib (JAB-21822) reported favorable safety and preliminary efficacy in advanced solid tumors (Jian et al., 2022; Shi et al., 2024), while olomorasib (LY3537982) exhibited pan-tumor activity in multiple KRAS G12C-mutant cancers (Heist et al., 2024). However, some inhibitors have encountered setbacks; clinical trials for JNJ-74699157 (ARS-3248) and LY34999446 were terminated due to toxicity concerns, and Novartis recently withdrew a Phase II study of JDQ443 (opnurasib), discontinuing its development (Cassier et al., 2023; Zhou et al., 2024). As research progresses, next-generation KRAS inhibitors with improved pharmacokinetics, stronger resistance profiles, and better combination potential are expected to refine the treatment landscape for KRAS G12C-mutant NSCLC.

### 3.2 Mechanism of KRAS G12C inhibitors resistance

**FIGURE 3:**
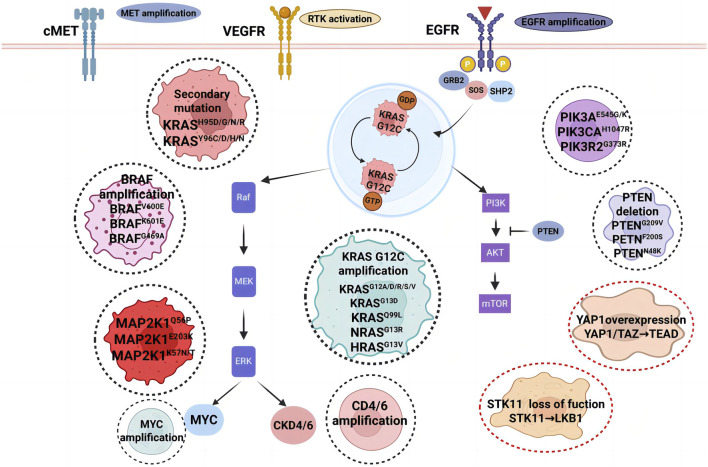
Resistance mechanisms to KRAS G12C inhibition. The black dashed circle highlights predominant acquired resistance mechanisms in KRAS-driven malignancies, encompassing KRASG12C gene amplification, oncogenic activation via mutations in KRAS, NRAS, and PIK3CA, along with secondary mutations in the KRAS inhibitor-binding pocket. The red dashed circle denotes adaptive resistance pathways associated with cell state transitions. Abbreviations: VEGFR, vascular endothelial growth factor receptor; EGFR, epidermal growth factor receptor; PI3K, phosphoinositide 3-kinase.

As KRAS G12C inhibitors advance in clinical research, they have provided significant therapeutic benefits for patients with KRAS G12C-mutant NSCLC. However, their long-term efficacy is often limited by resistance mechanisms, which can be classified into intrinsic (primary) resistance and acquired resistance (Figure 3). Despite early optimism regarding the development of KRAS G12C inhibitors, resistance to these therapies has become a major challenge, underscored by consistently short durations of response across pivotal trials. A direct comparison of time-to-resistance using median progression-free survival (mPFS) as a proxy reveals that both agents fail to meet the anticipated 6-month efficacy benchmark. In sotorasib trials, mPFS was 6.8 months in CodeBreaK 100 and 5.6 months in CodeBreaK 200. For adagrasib, mPFS was 5.4 months in KRYSTAL-1 and 5.49 months in KRYSTAL-12. These findings demonstrate that median time to disease progression remains approximately 5–6 months for both agents, highlighting the urgent need to address the rapid emergence of resistance (Lee et al., 2024). Primary resistance occurs when tumor cells exhibit low dependency on KRAS signaling, reducing their sensitivity to KRAS inhibition. Acquired resistance, on the other hand, emerges after treatment initiation and can result from selection-driven expansion of pre-existing KRAS subclones, bypass signaling activation, or histological transformations, all of which enable tumor cells to evade targeted inhibition (Kim et al., 2020; Rosell et al., 2021; Huang et al., 2021). Both resistance types may coexist in patients undergoing KRAS G12C-targeted therapy, further complicating treatment strategies (Vasan et al., 2019).

#### 3.2.1 Primary resistance (intrinsic resistance)

KRAS is a key driver of the RTK-RAS-MAPK signaling axis, and feedback reactivation from both upstream receptor tyrosine kinases (RTKs) and downstream mitogen-activated protein kinases (MAPKs) can contribute to primary resistance (Bahar et al., 2023). Preclinical studies suggest that primary resistance significantly influences the variability in clinical responses to KRAS G12C inhibitors. Some KRAS-mutant cells exhibit low reliance on KRAS activation for survival, enabling them to evade targeted therapy. Studies have shown that key downstream effectors, such as ERK and AKT, can remain active even after KRAS inhibition, suggesting alternative mechanisms of tumor survival (Vasan et al., 2019; Adachi et al., 2020; Misale et al., 2019; Singh et al., 2009). The PI3K-AKT-mTOR pathway, which plays a crucial role in cellular signaling, is not solely dependent on RAS activation, implying that tumor cells may activate alternative pathways to bypass KRAS inhibition (Ning et al., 2022). Additionally, cross-regulation between PI3K-AKT-mTOR and RAF-MEK-ERK pathways provides escape routes that allow cancer cells to maintain oncogenic signaling despite KRAS blockade (Misale et al., 2019; Ersahin et al., 2015; Heavey et al., 2014). Another major factor contributing to primary resistance is the presence of co-existing genetic alterations that enhance the GTP-bound state of KRAS, thereby reducing drug efficacy. Mutations that accelerate nucleotide exchange or impair GTPase activity have been linked to resistance (Garraway and Jänne, 2012; Lito et al., 2016). Notably, clinical trials have reported that patients with KEAP1 co-mutations frequently exhibit poor outcomes with KRAS G12C inhibitors, a finding supported by real-world data from KRYSTAL-1 and CodeBreaK 100 trials (Thummalapalli et al., 2023; Althoff et al., 2023).

#### 3.2.2 Acquired resistance

It has been postulated that acquired resistance to non-KRAS G12C mutations may be present at baseline and become more prominent during treatment with KRAS G12C inhibitors (Marinelli et al., 2021), reflecting a selection and dominance of resistant subclones. Currently, the acquired resistance to KRAS G12C inhibitors is primarily driven by on-target resistance (subclonal KRAS escape), off-target resistance (bypass pathway activation), and histological transformation (Awad et al., 2021).

##### 3.2.2.1 On-target mechanisms of acquired resistance

One of the most common acquired resistance mechanisms involves the selection and dominance of secondary KRAS mutations (termed “subclonal KRAS escape”) or increased KRAS G12C copy numbers, rendering tumors refractory to initial KRAS G12C inhibition. Secondary mutations within the Switch II pocket disrupt drug binding, ultimately reducing inhibitor efficacy (Tanaka and Ebi, 2024). For example, the KRYSTAL-1 trial identified multiple secondary KRAS mutations (G12D, G12R, G12V, G12W, Q61H, and Y96D) in patients who developed resistance to adagrasib, demonstrating how tumors adapt to evade inhibition (Awad et al., 2021; Singhal et al., 2024). Studies using resistant cell models have confirmed that different secondary KRAS mutations can affect sensitivity to various inhibitors, highlighting the complexity of overcoming KRAS G12C resistance. In an *in vitro* study by Koga et al., 76% of Sotorasib-resistant clones and 97% of Adagrasib-resistant clones developed secondary KRAS mutations, further supporting the role of on-target resistance in treatment failure (Koga et al., 2021).

##### 3.2.2.2 Off-target mechanisms of acquired resistance

Bypass signaling activation is another major contributor to acquired resistance, allowing tumor cells to circumvent KRAS inhibition by reactivating upstream or downstream pathways. One common mechanism involves RTK upregulation, where signaling through MET, HER2, or FGFR activates parallel pathways such as PI3K-AKT-mTOR and RAF-MEK-ERK, maintaining oncogenic signaling despite KRAS inhibition. For instance, Sotorasib-resistant cells have been shown to exhibit MET amplification, leading to persistent ERK phosphorylation, a resistance mechanism that can be reversed through MET inhibition (Addeo et al., 2021; Suzuki et al., 2021). Clinical analysis of pre- and post-treatment tumor samples from sotorasib-treated patients has revealed additional mutations in NRAS, BRAF, and EGFR, further underscoring the complexity of bypass resistance (Zhao et al., 2021). Another key mechanism is RAS-MAPK pathway reactivation, where mutant KRAS can activate wild-type RAS isoforms, leading to adaptive reactivation of oncogenic signaling. This resistance mechanism can be potentially overcome by combining SHP2 inhibitors (e.g., TNO155, RMC-4630) with KRAS G12C inhibitors, as demonstrated in preclinical and early-phase clinical studies (Jian et al., 2022; Singhal et al., 2024; Ryan et al., 2020; Wang et al., 2023). The KRYSTAL-1 trial also highlighted several alternative bypass resistance pathways, including activating mutations in NRAS, BRAF, MAP2K1, and RET, as well as oncogenic fusions involving ALK, RET, BRAF, RAF1, and FGFR3. Loss-of-function mutations in NF1 and PTEN, which act as negative regulators of oncogenic signaling, have also been implicated in acquired resistance to KRAS G12C inhibitors (Awad et al., 2021).

##### 3.2.2.3 Histological transformation

Another notable resistance mechanism is tumor histological transformation, in which lung adenocarcinomas evolve into alternative histological subtypes, such as squamous cell carcinoma (SCC) or neuroendocrine tumors, effectively escaping KRAS-targeted therapy. Studies have shown that under KRAS inhibition, lung adenocarcinoma cells can transdifferentiate into alveolar type 1 (AT1) cells, allowing cancer cells to survive despite targeted therapy (Li et al., 2024c; Tong et al., 2024). Epithelial-mesenchymal transition (EMT), a key process in tumor plasticity, has also been associated with resistance in KRAS G12C-mutant cell lines, further complicating treatment strategies (Adachi et al., 2020).

##### 3.2.2.4 Other factors associated with acquired resistance to KRAS G12C inhibitors

In addition to genetic alterations and pathway reactivation, several epigenetic and signaling adaptations have been linked to resistance. Re-expression of KRAS following inhibitor treatment has been observed in preclinical models, mediated by Hedgehog signaling and Aurora kinase A (AURKA), both of which promote KRAS reactivation (Zhang et al., 2023; Lee et al., 2024). Other pathways implicated in adaptive resistance include AXL signaling, tissue factor (TF) expression, and YAP1/TAZ-TEAD signaling, which contribute to tumor cell survival and immune evasion (Morimoto et al., 2024; Zhang et al., 2024; Edwards et al., 2023). Furthermore, disruption of protein homeostasis networks, such as the heat shock response and unfolded protein response, may also play a role in resistance, emphasizing the need for a multifaceted approach to overcoming KRAS G12C inhibitor resistance (Lv et al., 2023).

KRAS G12C inhibitors have marked a significant breakthrough in targeted therapy for NSCLC; however, their clinical efficacy remains hindered by intrinsic and acquired resistance mechanisms. While on-target resistance through secondary KRAS mutations remains a major challenge, off-target resistance involving bypass pathway activation and histological transformation further complicates long-term treatment efficacy. Combination strategies targeting multiple pathways, innovative next-generation KRAS inhibitors, and precision medicine approaches will be essential for overcoming these barriers and improving patient outcomes.

### 3.3 Drug combination therapy

**FIGURE 4:**
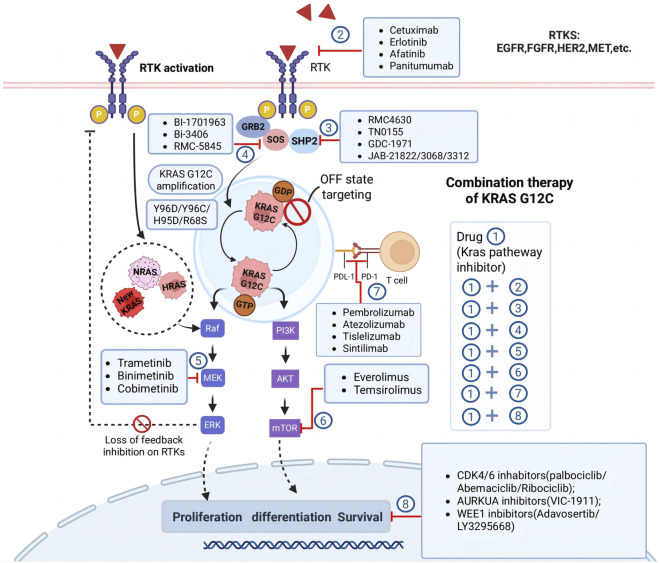
Combination therapy strategies for KRASG12C inhibition. The figure highlights several approaches to overcome resistance and enhance therapeutic efficacy, involve targeting feedback activation of receptor tyrosine kinases (RTKs), inhibiting downstream effectors such as MEK, mTOR, and CDK4/6, targeting emerging resistance mechanisms like KRAS G12C amplification or secondary mutations in other RAS family members (NRAS, HRAS). The diagram also emphasizes the potential of combining KRAS G12C inhibitors with immunotherapies or other novel agents like AURKA and WEE1 inhibitors to achieve synergistic effects. Abbreviations: RTK, receptor tyrosine kinase; EGFR, epidermal growth factor receptor; VEGFR, vascular endothelial growth factor receptor; PI3K, phosphoinositide 3-kinase.

Long-term monotherapy with KRAS G12C inhibitors, such as sotorasib and adagrasib, has demonstrated limited durability due to the emergence of primary and acquired resistance. As discussed, both agents exhibit a progression-free survival (PFS) of less than 6 months, highlighting the need for combination strategies to enhance efficacy and prevent resistance (Yaeger et al., 2023). The diverse resistance mechanisms associated with KRAS G12C inhibitors necessitate the development of combination therapies targeting multiple pathways to provide sustained clinical benefits (Figure 4). Combination approaches aim to block bypass signaling pathways, enhance immune responses, and disrupt the adaptive resistance mechanisms that arise during KRAS G12C-targeted treatment.

#### 3.3.1 Combining vertical pathway therapeutic strategies

The Ras gene is a downstream molecule of the EGFR gene, which can inhibit the Ras signaling pathway through endogenous ligands that competitively bind to the EGFR tyrosine kinase inhibitor (EGFR-TKI), thereby exerting anticancer effects. Osimertinib, a third-generation EGFR-TKI, is highly effective in patients with NSCLC who have acquired the EGFR T790M mutation (Tsuboi et al., 2023). However, the regulation of EGFR signaling could not inhibit the mutant Ras gene in the sustained activation state, so EGFR-TKI was ineffective against KRAS-mutated tumors. However, progression-free survival and overall survival have been significantly longer in patients with NSCLC after EGFR-TKI plus chemotherapy than with EGFR-TKI alone. Targeting the upstream signal of Ras generally has off-target effects and unsatisfactory therapeutic effect, but it is expected that the therapeutic effect can be improved if EGFR-TKI is combined with other treatments. A recent subgroup of studies in CodeBreaK101, a Phase I/II study evaluating sortorexab in combination with different regimens, recently reported that the combination of sotorexil, pemetrexed, and carboplatin achieved a proven ORR of 65% and 42%, respectively, in the first- and second-line treatments, of 58 patients with advanced NSCLC with KRAS G12C (Li B. T. et al., 2024).

Given that KRAS functions downstream of receptor tyrosine kinases (RTKs) such as EGFR, MET, and HER2, inhibiting these upstream regulators in combination with KRAS G12C inhibitors has emerged as a promising strategy to enhance therapeutic efficacy and counteract resistance. Preclinical studies have demonstrated that RTK-mediated feedback activation plays a critical role in limiting the efficacy of KRAS G12C inhibitors, making dual inhibition an attractive approach to prevent adaptive resistance (Ryan et al., 2020). While first-generation EGFR tyrosine kinase inhibitors (TKIs) were largely ineffective in KRAS-mutant NSCLC due to constitutive RAS activation, emerging evidence suggests that combining KRAS G12C inhibitors with EGFR-targeted therapies may enhance response rates.

The CodeBreaK 101 trial (NCT04185883) evaluated sotorasib in combination with afatinib (an irreversible EGFR/HER2 inhibitor) in KRAS G12C-mutant NSCLC, reporting objective response rates (ORRs) of 33% in cohort 1% and 34.8% in cohort 2, supporting the potential synergy between EGFR and KRAS inhibition (Moll et al., 2018; David et al., 2021). Another strategy targets guanine nucleotide exchange factor 1 (SOS1) and Src homology 2 domain-containing protein tyrosine phosphatase 2 (SHP2). SOS1 is a guanine nucleotide exchange factor that controls the activation of the KRAS G12C protein through its nucleotide-exchanging function (Vigil et al., 2010). *In vitro* study indicated that the combination of SOS1 inhibitors and MEK inhibitors could overcome acquired resistance from secondary mutations to KRAS G12C inhibitors in NSCLC (Koga et al., 2021). SHP2, a critical phosphatase that facilitates RTK signaling and plays a key role in activating wild-type RAS proteins, which can mediate bypass resistance to KRAS G12C inhibitors. Early-stage SHP2 such as RMC-4630 in combination with cobimetinib (MEK inhibitor) in NSCLC has no significant advantage compared to monotherapy. In clinical trials of solid tumors, patients often do not ultimately benefit due to efficacy and toxicity issues (Falchook et al., 2022). Novel SHP2 inhibition has been shown to prevent adaptive reactivation of the MAPK pathway, thereby improving the durability of KRAS-targeted therapy. The KontRASt-01 clinical trial is currently assessing the SHP2 inhibitor TNO155 in combination with KRAS G12C inhibitors, with preliminary data suggesting enhanced tumor suppression compared to monotherapy (Negrao et al., 2023a). Similarly, a Phase I/II study evaluating glecirasib (JAB-21822) in combination with the SHP2 inhibitor JAB-3312 reported a 77.8% ORR and a 92.6% disease control rate (DCR) in advanced solid tumors, though grade ≥3 treatment-related adverse events (TRAEs) occurred in 41.9% of patients (Jun et al., 2024).

Targeting downstream components of the RAF-MEK-ERK pathway has also emerged as a viable vertical inhibition strategyKRAS G12C inhibitors may fail to fully suppress MAPK signaling due to compensatory pathway activation, necessitating the use of MEK inhibitors to sustain inhibition. In the CodeBreaK 101 trial, the combination of sotorasib and trametinib achieved a partial response rate of 20% and a DCR of 87%, with 67% of patients who had previously received KRAS G12C inhibitors maintaining disease control (Ramalingam, 2021). Additionally, the RAMP203 Phase Ib/II trial (NCT05074810) is investigating the combination of avutometinib, a dual RAF/MEK inhibitor, with sotorasib, showing preliminary evidence of enhanced anti-tumor activity and delayed resistance development (CAPELLETTO et al., 2022; Awad et al., 2023).

Monoclonal antibodies targeting EGFR have also been explored in combination with KRAS G12C inhibitors. The latest results from the KROCUS study, combining fulzerasib (GFH925/IBI351) with cetuximab, were released at the 2025 European Lung Cancer Conference (ELCC). Reporting an ORR of 80% and a DCR of 100% in first-line KRAS G12C-mutated NSCLC, with TRAEs occurring in 87.2% of patients, though no grade 4 or 5 toxicities were observed (Majem et al., 2025). These findings underscore the potential of targeting multiple nodes within the RAS-MAPK pathway to achieve more durable treatment responses.

Despite the promising efficacy observed with vertical inhibition strategies, several challenges remain, including increased toxicity, dose-limiting adverse effects, and the need for biomarker-driven patient selection. Combinations involving SHP2 inhibitors have been associated with gastrointestinal and hematologic toxicities, while MEK inhibitors frequently lead to rash, diarrhea, and fatigue, which may limit tolerability. The identification of predictive biomarkers, such as co-occurring genetic alterations (e.g., STK11, KEAP1, NF1 mutations), RTK expression levels, and MAPK pathway activity, will be critical for refining patient selection and optimizing combination therapy. Ongoing clinical trials and translational studies will be essential in determining the most effective combination regimens while balancing efficacy and safety profiles for patients with KRAS G12C-mutant NSCLC.

#### 3.3.2 Parallel pathway therapeutic strategies

Argeting parallel oncogenic pathways has emerged as a critical strategy to overcome resistance to KRAS G12C inhibitors and enhance therapeutic efficacy. Tumor cells frequently develop bypass resistance mechanisms that sustain survival and proliferation despite KRAS inhibition, primarily through activation of alternative signaling cascades such as the PI3K-AKT-mTOR, JAK-STAT, and YAP1/TEAD pathways (Xue et al., 2020; Molina-Arcas et al., 2019). These compensatory mechanisms enable tumors to evade the effects of KRAS-targeted therapies, necessitating combination approaches to improve clinical outcomes.

The PI3K-AKT-mTOR pathway plays a fundamental role in cellular metabolism, proliferation, and survival and is frequently upregulated in KRAS-mutant cancers. Its activation can occur independently of KRAS signaling through receptor tyrosine kinase (RTK) upregulation or PTEN loss, creating an escape mechanism for tumor cells following KRAS inhibition (Tanaka et al., 2021). Preclinical studies have demonstrated that co-targeting KRAS G12C and the PI3K-AKT-mTOR pathway enhances tumor suppression and delays resistance. Notably, the combination of adagrasib with the mTOR inhibitor everolimus has shown significant tumor regression in preclinical models, while early clinical studies are evaluating the efficacy of PI3K inhibitors (e.g., buparlisib) and AKT inhibitors (e.g., capivasertib) in combination with KRAS G12C inhibitors (Molina-Arcas et al., 2019; Yaeger, 2025).

Another key survival pathway implicated in resistance is the JAK-STAT signaling cascade, which contributes to tumor immune evasion, inflammation, and proliferation. Persistent JAK-STAT activation allows cancer cells to bypass KRAS inhibition and sustain tumor growth (Cullis et al., 2018). Preclinical evidence suggests that blocking JAK-STAT signaling enhances the efficacy of KRAS G12C inhibitors by promoting tumor apoptosis and delaying resistance development. The combination of ruxolitinib (a JAK1/2 inhibitor) with KRAS G12C inhibitors is currently being investigated in clinical trials, with early results indicating improved response rates compared to monotherapy (Zdanov et al., 2016).

The YAP1/TEAD pathway, a major component of the Hippo signaling cascade, has also been identified as a key driver of KRAS inhibitor resistance. YAP1 activation promotes epithelial-to-mesenchymal transition (EMT), cancer stemness, and immune evasion, allowing tumors to maintain oncogenic signaling despite KRAS inhibition (Molina-Arcas and Downward, 2024). High YAP1 expression has been correlated with poor responses to KRAS-targeted therapy, suggesting that inhibition of this pathway may improve clinical outcomes. Based on the key role of YAP1 in resistance to KRAS inhibitors, preclinical studies are exploring the potential of combination therapies with YAP1 inhibitors such as verteporfin (Edwards et al., 2023; Yang et al., 2024).

Targeting parallel pathways represents a promising approach to counteracting resistance in KRAS-mutant NSCLC by disrupting compensatory survival mechanisms. While early preclinical and clinical results suggest that co-targeting PI3K-AKT-mTOR, JAK-STAT,and YAP1/TEAD pathways may enhance the efficacy of KRAS G12C inhibitors, further research is necessary to optimize these combination strategies. Ongoing clinical trials will be instrumental in identifying the most effective regimens that maximize therapeutic benefits while minimizing toxicity, ultimately improving patient outcomes in KRAS G12C-mutant cancers.

#### 3.3.3 Combined immunization strategies

Immunotherapy stands at the forefront of cancer research, with a burgeoning number of clinical trials dedicated to evaluating the therapeutic potential of anti-PD-1/PD-L1 immune checkpoint inhibitors. Extensive clinical findings suggest that immune checkpoint inhibitors (ICIs) have a positive impact on the survival of patients suffering from advanced cancer (Addeo et al., 2019). *In vitro* studies have revealed that KRAS-mutant cancers exhibit immunosuppressive properties, leading to the formation of regulatory T cells (Li et al., 2022a; Garassino et al., 2025). Oncogenic KRAS signaling significantly alters the gene expression profile of cancer cells, resulting in the overproduction of immunomodulatory cytokines and chemokines such as interleukin-10 (IL-10) and transforming growth factor beta (TGF-β). These changes may contribute to tumor immune evasion by suppressing immune effector cells and increasing the levels of immunomodulatory cells within the tumor microenvironment. Additionally, the immune response in KRAS-mutant tumors can be further influenced by mutations in tumor suppressor genes, including p53, LKB1/STK11, and KEAP1, which drive immune escape (Burns et al., 2024). Studies by Canon, Briere, and colleagues explored the combination of anti-PD-1 checkpoint inhibitors with KRAS G12C inhibitors AMG-510 or MRTX849 in mouse models with the CT-26(Colon Tumor #26)KRAS G12C mutation. The combination with anti-PD-1 antibodies resulted in complete tumor regression in most mice (Rojas et al., 2024).

The Phase Ib CodeBreak 100/101 study (NCT04185883) examined the safety and efficacy of combining the KRAS G12C inhibitor sotorasib with PD-L1 inhibitors (pembrolizumab or atezolizumab). Results from the 2022 WCLC showed an overall efficacy of 29% with a disease control rate of 83%. The median duration of response was 17.9 months. However, this combination may lead to hepatotoxicity. The combination of Sotorasib with a PD-(L)1 inhibitor may induce more and more severe hepatotoxicity. The lead-in cohort has demonstrated durable clinical activity and a better safety profile compared with a concurrent dosing regimen (Li et al., 2022a). Lowering the dose and infusion of Sotorasib is preferred to achieve better tolerability. KRYSTAL-7 is a Phase II study investigating Adagrasib in combination with immunotherapy for the first-line treatment of KRAS G12C-mutated advanced NSCLC. The primary endpoint of the study was objective response rate (ORR) as assessed by a blinded independent review center (BICR) (RECIST v1.1). In the latest report of the KRYSTAL-7 trial (evaluable number = 53), Adagrasib and Pembrolizumab (PD-1 inhibitors) had an ORR of 59% and a DOR of 26.3 months (NCT04613596). At median follow-up beyond 22 months, median progression-free survival (PFS) was 27.7 months, and 18-month overall survival (OS) was 62%. In terms of safety, the KRYSTAL-7 study had an incidence of grade 3 or higher TRAEs in combination with pembrolizumab in 68%, respectively, the incidence of TRAEs leading to discontinuation was low (Garassino et al., 2025).

A recent study of LY3537982 combined with pembrolizumab in 30 patients with KRAS G12C mutations showed promising results. With a median follow-up of 6 months, the overall response rate was 63% and the disease control rate was 93%. For patients with PD-L1 expression ≥50%, the response rate was 75%, compared to 56% for those with lower or unknown PD-L1 expression. In first-line treatment patients, the response rate was 78% and the disease control rate was 100% (Burns et al., 2024). MK-1084, a selective KRAS G12C-GDP inhibitor developed by Merck & Co., Inc., has also shown promising anti-tumor activity. In a Phase 1 study presented at the 2024 ESMO Congress, MK-1084 combined with pembrolizumab demonstrated an objective response rate of 70% and a disease control rate of 87% at doses of 25–200 mg daily. Higher doses (≥400 mg) resulted in an objective response rate of 75% and a disease control rate of 75% (Rojas et al., 2024). A summary of studies on drugs with KRAS G12C inhibitory activity in combination with immunosuppressants or other drugs can be found in Table 2.

**TABLE 2:** A summary of current trials assessing KRAS G12C inhibitors in combination with other Inhibitors/Immunotherapies/Chemotherapies.

Drug	Trial name	NCT number	Sponsor	Phase	Combination therapy	References
Sotorasib (AMG 510)	CodeBreaK 101	NCT04185883	Amgen	I/II	Trametinib (MEK inhibitor)	Miyashita et al. (2024)
					TNO155 (SHP2 inhibitor)	
					Everolimus (mTOR inhibitor)	
					Palbociclib (CDK4/6 inhibitor)	
					Afatinib (EGFR-TKI)	David et al. (2021)
					Pembrolizumab (PD-1 antibody)	
					Atezolizumab (PD-L1 antibody)	Amgen et al. (2023a)
					Chemotherapy (CBDCA, PEM, DTX)	
					RMC-4630 (SHP2 inhibitor)	Falchook et al. (2022)
	CodeBreaK 100	NCT03600883	Amgen	I/II	PD-1 or PD-L1 antibodies	Li et al. (2021)
	CodeBreaK 202	NCT05920356	Amgen	III	Carboplatin and pemtrexed + Sotorasib/Pembrolizumab	Amgen et al. (2023b)
	KRYSTAL 1	NCT03785249	Mirati	I/II	Afatinib (EGFR-TKI)	Negrao et al. (2023)
					Pembrolizumab (PD-1 antibody)	
Adagrasib (MRTX849)	KRYSTAL 7	NCT04613596	Mirati	II/III	Pembrolizumab (PD-1 antibody)	Mirati Therapeutics Inc (2023)
		NCT04330664	Mirati	I/II	TNO155 (SHP2 inhibitor)	Sabari et al. (2021)
JDQ443	KontRASt-01	NCT04699188	Novartis	I/II	TNO155 (SHP2 inhibitor)	Novartis Pharmaceuticals (2021)
					Tislelizumab (PD-1 antibody)	
LY3537982 (olomorasib)	LOXO-RAS-20001	NCT04956640	Eli Lilly	I	Abemaciclib (CDK4/6 inhibitor)	Murciano-Goroff et al. (2023)
					Erlotinib (EGFR-TKI)	
					Sintilimab (PD-1 antibody)	
					Temuterkib (ERK inhibitor)	
					LY3295668 (Aurora kinase inhibitor)	
					Cetuximab (EGFR antibody)	
		NCT04956640			pembrolizumab	Burns et al. (2024)
GDC-6036 (Divarasib)	KRAScendo-170 Lung	NCT04449874	Hoffmann-La Roche	I	Erlotinib (EGFR-TKI)	Sacher et al. (2022)
					GDC-1971 (SHP2 inhibitor)	
					Bevacizumab (VEGF antibody)	
		NCT05789082	Hoffmann-La Roche	​	pembrolizumab ± platinum-based chemotherapy and pemetrexed	Skoulidis et al. (2024)
MK-1084	​	NCT05067283	Merck Sharp & Dohme	I	Pembrolizumab (PD-1 antibody)	Rojas et al. (2023)
D-1553 (Garsorasib)		NCT05492045	InventisBio	I/II	immunotherapy/targeted	Li et al. (2024b)
		NCT06166836		I/II	Ifebemtinib (IN10018,FAK inhibitor)	Song et al. (2024)
Fulzerasib (GFH925/IBI351)	KROCUS	NCT05756153	GenFleet Therapeutics	II	cetuximab	Majem et al. (2025)
JAB-21822 (Glecarisib)	​	NCT05288205	Jacobio	I/II	JAB-3312	Jun et al. (2024)

NCT: national clinical trial; EGFR-TKI: EGFR-tyrosine kinase inhibitor; CBDCA: carboplatin; PEM: pemetrexed; DTX: docetaxel.

#### 3.3.4 Other joint strategies

The CodeBreaK 101 Phase IB trial evaluated the safety and efficacy of combining sotorasib with carboplatin and pemetrexed in patients with KRAS G12C-mutant advanced NSCLC (Clarke et al., 2023). Recent results from the CodeBreak 101 Global study demonstrated encouraging clinical activity. In first-line NSCLC, the combination achieved an overall response rate (ORR) of 65% and a disease control rate (DCR) of 100%. In second-line or later settings, the ORR was 42% and the DCR was 84%. Additionally, the combination showed a median progression-free survival (PFS) of 11.9 months in the PD-L1-negative population (Li B. T. et al., 2024). These results have led to the initiation of the Phase II SCARLET trial and the Phase III CodeBreaK 202 trial (NCT05920356), which aims to compare sotorasib or pembrolizumab in combination with chemotherapy in first-line treatment for PD-L1-negative patients (Barlesi et al., 2024). The final analysis of the SCARLET study was introduced in ASCO 2024, indicating positive ORR and PFS results with manageable toxicity. The results of the SCARLET study showed that sortoraxib in combination with chemotherapy demonstrated an objective response rate of up to 88.9% and a median OS of 20.6 months in KRAS G12C-mutated advanced non-squamous non-small cell lung cancer (Yoshioka et al., 2024). KRAS-MAPK signaling is a key driver of cell cycle transition in the G1-S phase, thus, targeting cell cycle regulators, particularly those that drive the G1-S phase transition, shows promise as a co-targeting approach. Cyclin-dependent kinases 4 and 6 (CDK4/6) are pivotal for facilitating the transition from the G1 to S phase of the cell cycle. Their inactivation halts cell cycle progression at G0/G1. FDA-approved several related CDK4/6 inhibitors (such as Palbociclib, Ribociclib, and Abemaciclib) for use in combination with letrozole for the treatment of hormone receptor-positive advanced breast cancer and other cancers. In addition, combinations of CDK4/6 inhibitors with other targeted therapies may help overcome primary or secondary treatment resistance (Lv et al., 2024). Preclinical studies have explored this approach by investigating the combination of KRAS G12C inhibitors with Aurora kinase inhibitors (AURKA, AURKB, AURKC) and mitotic checkpoint kinase WEE1 inhibitors (Bagnyukova et al., 2024; Fukuda et al., 2024). Combining these cell cycle inhibitors with KRAS inhibitors may enhance therapeutic outcomes.

## 4 Challenges and future perspectives

The development of KRAS G12C inhibitors has marked a significant milestone in the treatment of KRAS-mutant non-small cell lung cancer (NSCLC), providing targeted therapeutic options for a subset of patients who previously had limited treatment choices. The approval of sotorasib and adagrasib has demonstrated the feasibility of targeting KRAS, leading to meaningful clinical benefits. However, despite these advances, intrinsic and acquired resistance remains a significant challenge, limiting the durability of responses in most patients (O'Sullivan et al., 2023; Shaverdashvili and Burns, 2025). The emergence of secondary KRAS mutations, bypass pathway activation, and histological transformation underscores the need for novel therapeutic strategies to enhance and sustain treatment efficacy (Singhal et al., 2024; Addeo et al., 2021; Suzuki et al., 2021; Zhao et al., 2021).

To address these challenges, combination therapy approaches are being actively explored, integrating KRAS G12C inhibitors with agents targeting RTKs (e.g., SHP2 inhibitors), downstream MAPK signaling (e.g., MEK inhibitors), parallel oncogenic pathways (e.g., PI3K-AKT-mTOR inhibitors), and immune checkpoint inhibitors to overcome resistance mechanisms and improve patient outcomes (Moll et al., 2018; David et al., 2021; Negrao et al., 2023a; Li et al., 2022a). However, treatment-related adverse events (TRAEs) remain a concern, particularly in combination regimens, emphasizing the need for optimized dosing strategies and biomarker-driven patient selection to balance efficacy and toxicity (Li et al., 2022a).

The suboptimal durability of current agents paradoxically strengthens, rather than diminishes, the rationale for intensified KRAS-directed research. Limited PFS confirms that transient KRAS inhibition is insufficient; sustained pathway suppression is imperative for long-term disease control. This precisely justifies the strategic pivot toward upfront combination regimens designed to prevent clonal selection of resistant subpopulations and suppress adaptive reprogramming. Importantly, advances in predictive biomarkers and real-time liquid biopsy now enable precise patient stratification and adaptive therapy, transforming combination strategies from empiric cocktails to optimized, personalized protocols. The therapeutic goal is clear: convert transient responses into durable remissions by addressing resistance before it emerges.

Future therapeutic directions will focus on next-generation KRAS inhibitors, including pan-KRAS inhibitors, allele-specific inhibitors (e.g., targeting KRAS G12D and G12V), and KRAS protein degraders, which hold promise for improving therapeutic outcomes beyond KRAS G12C-specific agents (Morimoto et al., 2024; Zhang et al., 2024; Edwards et al., 2023). Additionally, the integration of biomarker-driven precision medicine, liquid biopsy technologies for real-time monitoring, and AI-driven drug discovery is expected to revolutionize KRAS-targeted therapy, offering more personalized and effective treatment strategies (Zhang et al., 2023; Lee et al., 2024).

## 5 Conclusion

The therapeutic landscape for NSCLC has been considerably transformed by the advent of KRAS G12C-targeted therapies. However, to enhance long-term patient outcomes, it is imperative to address resistance mechanisms, refine combination approaches, and innovate next-generation inhibitors. Maximizing the potential of KRAS-targeted therapies in oncology will necessitate ongoing clinical research, robust translational studies, and the development of novel therapeutic strategies.
